# Immune checkpoint CD161/LLT1‐associated immunological landscape and diagnostic value in oral squamous cell carcinoma

**DOI:** 10.1002/cjp2.353

**Published:** 2024-01-17

**Authors:** Xinyang Hu, Yuexin Dong, Shixin Xie, Yuxian Song, Chenhang Yu, Yijia He, Zhiyong Wang, Qingang Hu, Yanhong Ni, Liang Ding

**Affiliations:** ^1^ Central Laboratory of Stomatology, Nanjing Stomatological Hospital, Affiliated Hospital of Medical School Nanjing University Nanjing PR China; ^2^ Department of Oral and Maxillofacial Surgery, Nanjing Stomatological Hospital, Affiliated Hospital of Medical School Nanjing University Nanjing PR China

**Keywords:** LLT1, CD161, oral squamous cell carcinoma, OSCC, immunotherapy, immune checkpoint, immune checkpoint inhibitors, PD‐1, PD‐L1, T cells, Foxp3

## Abstract

An active host adaptive response is characterized by the existence of programmed cell death protein 1 (PD‐1)^+^/IFN‐γ^+^ cytotoxic T cells and IFN‐γ‐induced PD‐L1^+^ tumor cells (TCs), which predicts high response rate to anti‐PD‐1/L1 therapy. Recently, CD161 and its ligand LLT1 (CLEC2D) have been identified as an emerging checkpoint for immunotherapy. Clarifying its heterogeneous clinical expression pattern and its immune landscape is a prerequisite for maximizing the response rate of CD161 blockade therapy in a specific population of oral squamous cell carcinoma (OSCC) patients. Here, we investigated the expression pattern of CD161/LLT1 and its association with major immunocytes (T cells, B cells, NK cells, and macrophages) by multiplex immunofluorescence, immunohistochemistry, and flow cytometry in 109 OSCC tissues and 102 peripheral blood samples. TCs showed higher LLT1 levels than tumor infiltrating lymphocytes (TILs), whereas CD161 was highly expressed in CD8^+^ T cells at the tumor front, which was decreased in paracancerous tissue. High expression of TC‐derived LLT1 (LLT1^TC^) conferred poor clinical outcomes, whereas higher CD161^+^ and LLT1^+^ TILs were associated with better prognosis. Meanwhile, patients with high LLT1^TC^ showed a decreased ratio of CD8^+^/Foxp3^+^ T cells *in situ*, but CD161^+^ TILs correlated with more peripheral CD3^+^ T cells. Interestingly, treatment of OSCC patients with nivolumab (anti‐PD‐1) could restore tumoral CD161/LLT1 signal. Furthermore, an OSCC subgroup characterized by high LLT1^+^ TCs and low CD161^+^CD8^+^ T cells showed fewer peripheral T cells and a higher risk of lymph node metastasis, leading to a shorter 5‐year survival time (29%). More LLT1^TC^ at the invasive front was another risk characteristic of exhausted T cells. In conclusion, in view of this heterogeneity, the LLT1/CD161 distribution pattern should be determined before CD161‐based immunotherapy.

## Introduction

Oral squamous cell carcinoma (OSCC) is one of the most common malignancies in the head and neck [1, 2]. However, traditional treatments can cause significant side effects and may not be effective for all patients [3, 4]. Recently, immunotherapy, including immune checkpoint inhibitors, has emerged as a promising treatment option for oral cancer [5, 6]. Immune checkpoint inhibitors, such as those that target programmed cell death protein 1 (PD‐1), have shown encouraging results since they were approved in 2014 for the treatment of various solid tumors [7]. An active host adaptive response is characterized by the existence of cytotoxic T cells and IFN‐γ‐induced programmed death‐ligand 1 (PD‐L1) expression in tumor cells (TCs), which predicts a high response rate to anti‐PD‐1/L1 therapy [8, 9]. We previously found that stromal IL‐33/ST2 signaling directly or indirectly enhanced PD‐L1‐mediated immune escape and OSCC progression. ST2^high^/PD‐L1^high^ OSCC patients might benefit more from anti‐PD‐1/L1 therapy. Therefore, patient stratification is a prerequisite for targeted therapy of PD‐1/L1 [9, 10].

CD161, a member of the killer cell lectin‐like receptor (KLR) family [also referred to as natural killer (NK) receptor‐P1A or KLRB1], is mainly expressed on both NK cells and T cells [11, 12]. Meanwhile, the *CLEC2D* gene is expressed in many hematopoietic cells and malignant cells, and *CLEC2D* variant 1 encodes LLT1 [13, 14]. LLT1 is the only isoform of the protein expressed on the cell surface, which binds to CD161 [11]; and the interaction between the CD161 receptor and LLT1 modulates the immune response and blocks killing of TCs expressing LLT1 [15].

Recently, CD161 has been extensively studied and characterized on multiple immune cells including CD4^+^, CD8^+^, and Foxp3^+^ T cells, which have high pro‐inflammatory capacity and rapid response in a variety of diseases, including Crohn's disease [16], childhood arthritis [17], and multiple sclerosis [18]. Studies have shown that the CD161/LLT1 interaction inhibits NK cell‐mediated cytotoxicity and IFN‐γ secretion, but its role in T cells remains controversial [19, 20]. The CD161/LLT1 interactions play a significant role in the immune system. On the other hand, high CD161 expression is more likely to be linked with improved survival such as in HPV16‐associated tumors [21] and lung cancer [22]. In contrast, a study revealed that low LLT1 expression in TCs was a favorable prognosis factor for HPV‐negative oropharyngeal squamous cell carcinoma [23]. Accumulating studies have also reported that blocking the CD161/LLT1 interaction enhances NK cell‐mediated lysis in various malignancies, including gliomas [24], triple‐negative breast cancer [25], and prostate cancer [26]. However, the correlation and influence of CD161/LLT1 in the OSCC immune microenvironment remain unclear.

In this study, we examined the expression patterns and colocalization of CD161/LLT1 in the OSCC tumor microenvironment (TME) and assessed their clinicopathological, prognostic value and association with major immunocytes (T cells, B cells, NK cells, and macrophages). Patient stratification combining CD161 and LLT1 helped us to identify patients with the worst clinicopathological performance and prognosis and to identify their immune status. Moreover, the relationship between CD161/LLT1 and anti‐PD‐1 therapy was also examined. Finally, the presence of TC‐derived LLT1 (LLT1^TC^) at the invasive front is another risk characteristic that predicts the phenotypic features of exhausted T cells.

## Materials and methods

### Patients and samples

**Figure 1:**
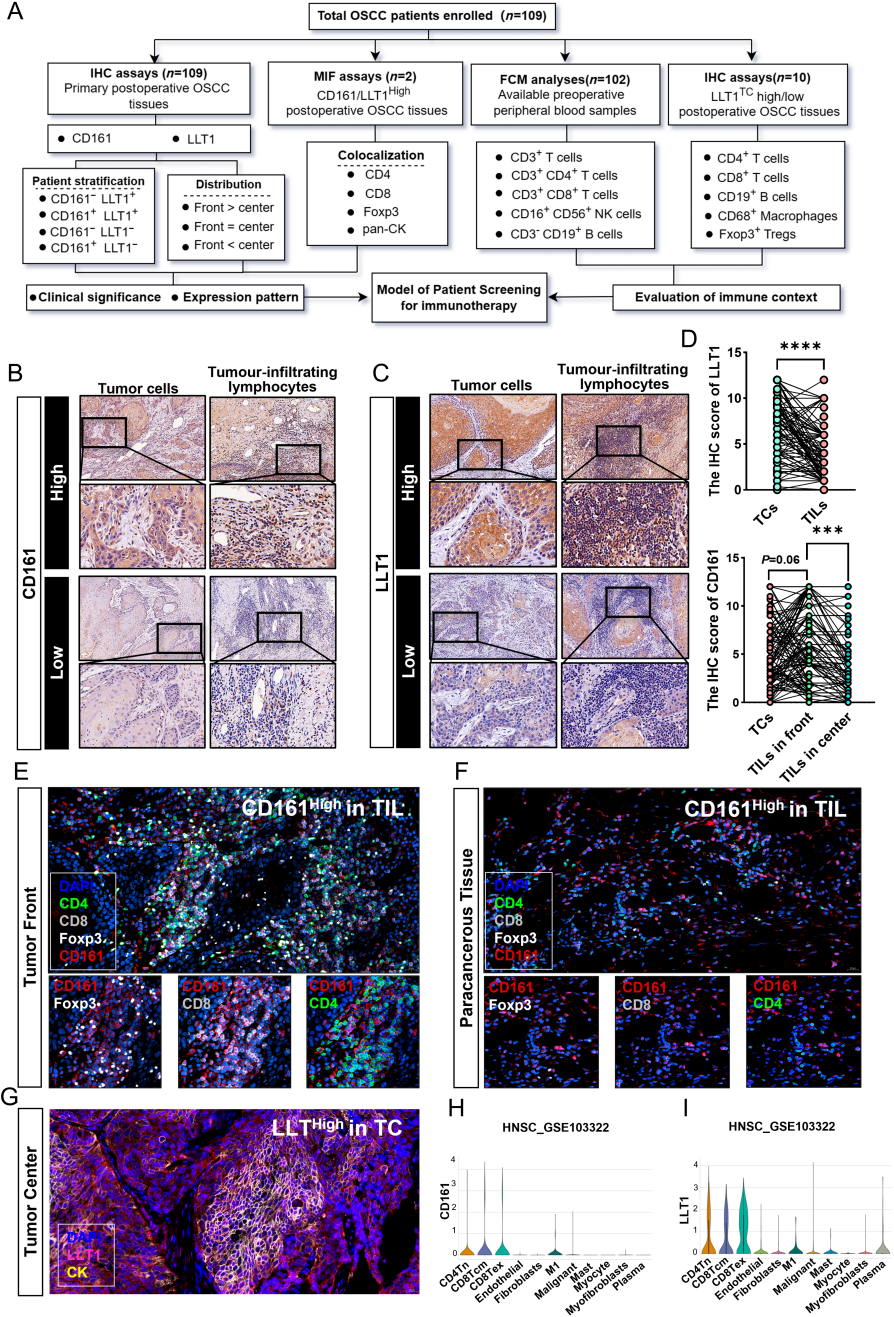
Expression patterns of CD161 and LLT1 in OSCC. (A) Flowchart for characterization of the patients enrolled in this study. (B) Representative images of IHC for low and high expression of LLT1 (upper magnification: ×200 and lower magnification: ×600). (C) Representative images of IHC for low and high expression of CD161 (upper magnification: ×200 and lower magnification: ×600). (D) Upper: the IHC score of LLT1 in TCs, and TILs from 109 OSCC patients. Lower: the IHC score of CD161 in TCs, and TILs at the invasive front and tumor center from 109 OSCC patients. (E) Representative images of MIF at the tumor front (magnification: ×200). (F) Representative images of MIF in paracancerous tissue (magnification: ×200). (G) Representative images of MIF at the tumor center (magnification: ×200). (H and I) Expression patterns of LLT1 and CD161 in HNSCC were validated using the single‐cell sequencing database (GSE103322) obtained from the TISCH2 single‐cell database. ***p ≤ 0.001, ****p ≤ 0.0001.

The patients enrolled in this study are briefly summarized in Figure 1A. One hundred nine primary patients with OSCC were retrospectively collected in this study. These unselected, nonconsecutive, and primary stage I–IV OSCC patients underwent curative resection between 2010 and 2017 at Nanjing Stomatology Hospital. All experiments were approved by the ethics committee of Nanjing Stomatology Hospital, Medical School of Nanjing University (NJSH‐1023NL‐03). Informed consent was obtained from the patients for the use of their tissues and data. The study was performed in accordance with the Declaration of Helsinki. Paraffin‐embedded OSCC tissue slices were obtained from the pathology department and used for immunohistochemistry (IHC). None of the patients received preoperative chemotherapy, radiotherapy, or other cancer‐related treatments. Patients with a history of systemic illness or missing survival data were excluded. Matched primary postoperative tumor samples and primary preoperative peripheral blood samples were collected from 102 of the 109 patients; for 7 patients, only tumor samples were available. Next, based on the IHC results of the 109 patients, we selected two cohorts of patients with characteristic CD161 or LLT1 expression for multiplex immunofluorescence (MIF) experiments and IHC evaluating the local immune microenvironment. The more detailed inclusion and exclusion criteria of patients were described in our previous study [27].

### Multiplex immunofluorescence

MIF staining was conducted at Nanjing Freethinking Biotechnology Co. Ltd. (Nanjing, PR China). Formalin‐fixed paraffin‐embedded sections were prepared as previously described [28]. The slides were first deparaffinized in xylene, rehydrated, and washed before boiling in Tris–EDTA buffer (50×, pH 9, Beyotime, Shanghai, PR China). After blocking the endogenous peroxidases by incubation in an antibody block for 10 min, the slide was detected in each round, including primary antibody incubation, secondary antibody incubation, and tyramide signal amplification visualization, followed by labeling the next antibody after epitope retrieval and protein blocking as described above. Sections were serially incubated with primary antibodies such as anti‐CD161 (67537‐1‐Ig, Proteintech, Wuhan, PR China), anti‐LLT1 (ab197341, Abcam, Cambridge, UK), anti‐CD4 (48274, Cell Signaling Technology, Beverly, MA, USA), anti‐CD8 (85336, Cell Signaling Technology), anti‐pan‐CK (ZM‐0464, ZSGB‐BIO, Wuxi, PR China), and anti‐Foxp3 (ab215206, Abcam, Shanghai, PR China). Finally, the slides were stained with 4′,6‐diamidino‐2‐phenylindole (Selleckchem, Shanghai, PR China) for nuclei and mounted with anti‐quenching sealing tablets.

### IHC and quantification

IHC was performed as previously described [28], and serial sections were incubated with primary antibodies such as anti‐CD161 (67537‐1‐Ig, Proteintech), anti‐LLT1 (ab197341, Abcam), anti‐CD4 (ZM‐0418, ZSGB‐BIO), anti‐CD8 (ZA‐0508, ZSGB‐BIO), anti‐CD19 (ZM‐0038, ZSGB‐BIO), anti‐CD68 (ZM‐0464, ZSGB‐BIO), and anti‐Foxp3 (ab253297, Abcam).

The IHC staining results of CD161 and LLT1 were independently and double‐blindly evaluated by two senior pathologists (Liang Ding and Qingang Hu) who were blinded to the patients' data, and the average values were calculated for further analysis. IHC staining was scored according to the percentage of positive cells and staining intensity. The percentage of stained cells was defined as 0 = 0–5%; 1 = 6–25%; 2 = 26–50%; 3 = 51–75%; and 4 = 75–100%. The staining intensity was defined as follows: 0 = negative staining; 1 = weak staining; 2 = moderate staining; and 3 = strong staining. The IHC score was calculated by multiplying the grade of the staining intensity by that of the staining percentage. High and low expression of LLT1 were defined as the median of IHC scores. The readout score of CD161 lymphocytes was subdivided into values for the tumor center and invasive margin, and high and low expression of CD161 lymphocytes were defined according to the median of the sum of these two values.

### Flow cytometry assay

Peripheral blood mononuclear cell (PBMC) samples were collected from patients' preoperative whole blood. For the analysis of PBMC cell subtypes, cells were collected, washed twice with phosphate‐buffered saline (PBS, Servicebio, Wuhan, PR China), and then suspended in 200‐μl PBS. BD Multitest™ CD3 FITC/CD8 PE/CD45 PerCP/CD4 APC reagent (Cat. No. 340499, BD Multitest™, San Jose, CA, USA) and BD Multitest™ CD3 FITC/CD16 PE + CD56 PE/CD45 PerCP/CD19 APC reagent (Cat. No. 340500, BD Multitest™) were used to enumerate the CD3^+^ T cells, CD3^+^ CD4^+^ T cells, CD3^+^ CD8^+^ T cells, CD19^+^ B cells, and CD56^+^ NK cells. This was followed by quantification using a fluorescence‐activated cell sorting Calibur instrument. All study participants provided informed consent.

### Gene correlation analysis in cBioportal

cBioPortal for Cancer Genomics (http://cbioportal.org) is a website for the exploration of multidimensional cancer genomics data, providing a readily understandable gene expression event [29]. We used cBioPortal to analyze the correlation among CD161, LLT1, and specific immune cell subset markers as well as specific immune checkpoint molecules in head and neck squamous cell cancer (HNSCC). Coexpression was calculated based on the cBioPortal's online instructions.

### Single‐cell RNA sequencing analysis of TISCH2

The Tumor Immune Single‐Cell Hub 2 (TISCH2) is a resource of single‐cell RNA‐seq data from human and mouse tumors, which enables comprehensive characterization of gene expression in the TME across multiple cancer types [30]. HNSC_GSE103322 single‐cell sequencing was used to validate the expression patterns of CD161 and LLT1 in head and neck tumors and their relationship with the clinical stage.

### Statistical analysis

SPSS 22.0 (IBM Corp, Armonk, NY, USA), GraphPad Prism 8.0 (Dotmatics, Boston, MA, USA), and Chiplot (https://www.chiplot.online/) were used for data analysis and graphical processing. Pearson's chi‐square test, Fisher's exact test, and the chi‐square test were used to compare clinicopathological features. The Mann–Whitney *U* test was used to compare the two groups. Survival analysis included overall survival (OS), metastasis‐free survival (MFS), and disease‐free survival (DFS), which were evaluated using Kaplan–Meier and log‐rank tests. Further multivariate analysis was carried out using the Cox proportional hazards regression model to determine the independent risk factors, adjusted hazard ratio (HR) and 95% confidence interval (CI) for OSCC. Coexpression of LLT1, CD161, immune cell markers, and immune checkpoint molecules was investigated by Pearson correlation analysis. All statistical tests were two‐sided, and *p* < 0.05 was considered to be significant.

## Results

### Expression patterns of CD161 and LLT1 in OSCC

Evaluating the distribution of CD161 and LLT1 in 109 OSCC tissues (Figure 1A), we found that CD161 and LLT1 were mainly expressed on tumor infiltrating lymphocytes (TILs) and TCs, respectively, both on the cell membrane and cytoplasm. Typical low and high expression of CD161 and LLT1 IHC staining are presented in Figure 1B,C. However, higher LLT1 levels were found in TCs of the tumor center, and TILs at the tumor front showed the highest CD161 expression (Figure 1D).

Considering the clinical significance of the CD161‐LLT1 signal, the expression of LLT1 by TCs may facilitate their escape from CD161^+^ immunocyte surveillance [24, 25]. Therefore, it is necessary to define the colocalization of other cellular signature markers with CD161^+^ lymphocytes and LLT1^+^ malignant cells. Consequently, we conducted MIF analysis of LLT1 in pan‐cytokeratin (pan‐CK)^+^ OSCC cells and CD161 in different T cell subgroups, including CD4^+^ T cells, CD8^+^ T cells, and Foxp3^+^ Tregs. Among the TILs in the OSCC microenvironment, CD161 was mainly distributed on the surface of CD8^+^ T cells at the tumor front. The percentages of CD161^+^CD4^+^ T cells or CD161^+^Foxp3^+^ Tregs were relatively low. Interestingly, the count of CD8^+^ CD161^+^ T cells was decreased in paracancerous tissue (Figure 1E,F). The MIF results also showed high overlapping expression of pan‐CK and LLT1^TC^ (Figure 1G). The same expression pattern was confirmed using the TISCH2 single‐cell database. As shown in Figure 1H,I, CD161 was mainly expressed by tumor‐infiltrating CD4^+^ and CD8^+^ T cells, with negligible expression by TCs. However, in this HNSCC patient cohort (*n* = 18), lymphocytes had higher LLT1 expression compared with TCs, which was different from the findings in OSCC patients (*n* = 109).

### Association of CD161/LLT1 with clinicopathologic characteristics and prognosis

To further explore the role of CD161 and LLT1 in OSCC, we analyzed the correlation between the expression of CD161/LLT1 and clinicopathological features of 109 OSCC patients (supplementary material, Table S1). Higher tumor LLT1 expression was associated with a higher risk of lymph node metastasis (supplementary material, Figure S1A) and advanced TNM stage (supplementary material, Figure S1B), which was consistent with the online TISCH2 database results (supplementary material, Figure S1C). Moreover, we did not observe a statistically significant association between CD161 expression and clinicopathological features (Figure 1D and supplementary material, Table S1).

**Figure 2:**
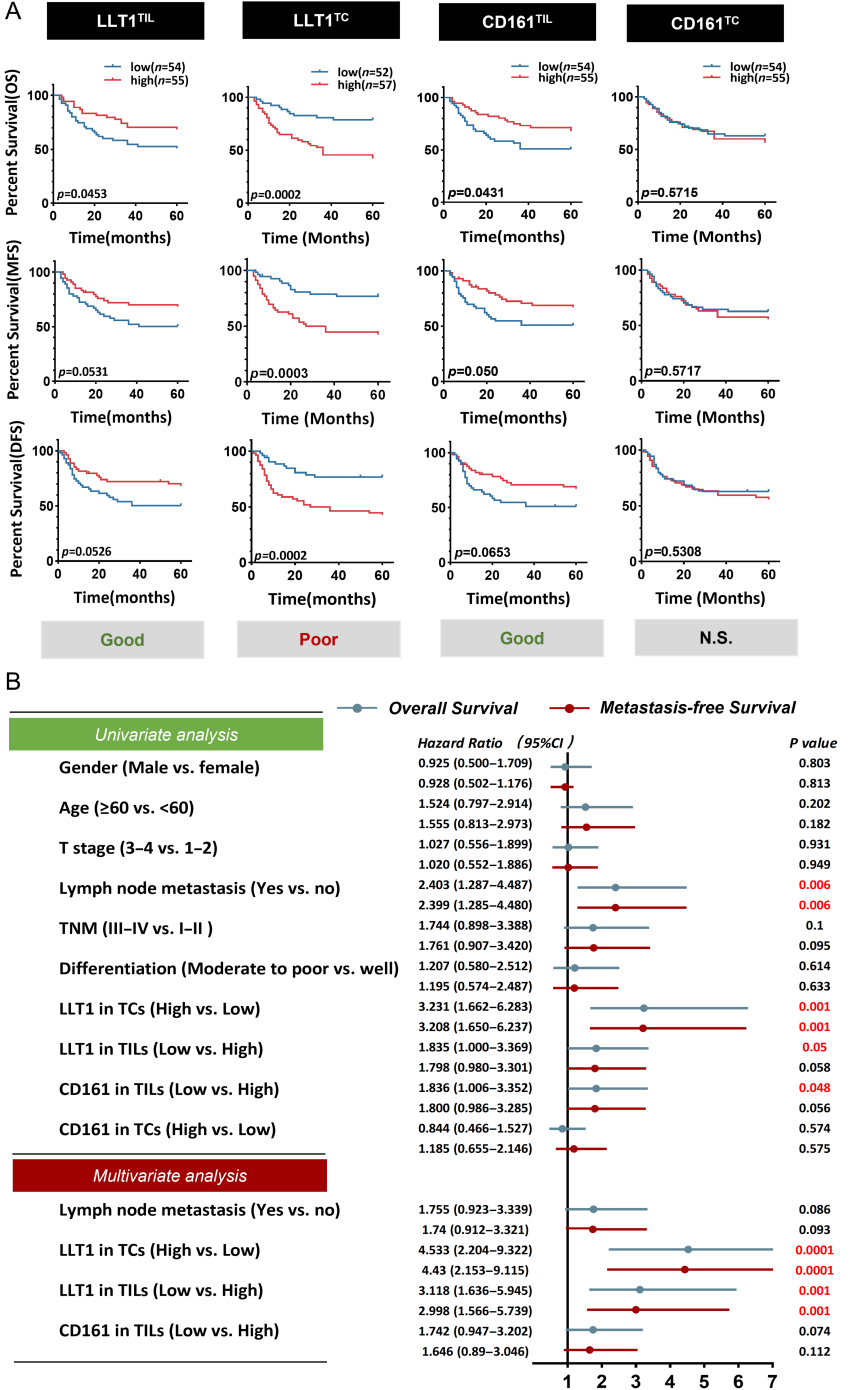
Association of CD161/LLT1 with prognosis. (A) Kaplan–Meier curves of OS, MFS, and DFS based on IHC classification of CD161 and LLT1 in TCs and TILs. The gray boxes at the bottom give the prognostic significance of high expression; log‐rank test was utilized for the analysis. (B) Univariate and multivariate Cox analyses of OS and MFS based on LLT1, CD161 expression, and clinicopathological factors. *p* values less than 0.05 are shown in red.

Next, we investigated the potential prognostic value of TC‐derived CD161 (CD161^TC^), lymphocyte‐derived CD161 (CD161^TIL^), LLT1^TC^, and lymphocyte‐derived LLT1 (LLT1^TIL^). Kaplan–Meier curve tests were conducted to compare the OS, MFS, and DFS between CD161/LLT1 high and low subgroups. The abundance of CD161^+^ TILs, but not CD161^+^ TCs, predicted better OS and MFS (*p* = 0.0431 and *p* = 0.050; Figure 2A). Conversely, high LLT1^TC^ levels were associated with shorter OS, MFS, and DFS (*p* = 0.0002, *p* = 0.0003, and *p* = 0.0002, respectively; Figure 2A). Interestingly, higher LLT1^TIL^ levels seemed to be associated with preferable OS (*p* = 0.0453; Figure 2A), which was consistent with previous reports [22, 23].

Subsequently, univariate and multivariate Cox regression analyses were performed (Figure 2B). Multivariate analyses suggested that LLT^TC^ (HR: 4.533, 95% CI: 2.204–9.322 for OS; and HR: 4.43, 95% CI: 2.153–9.115 for MFS) and LLT1^TIL^ (HR: 3.118, 95% CI: 1.636–5.945 for OS; and HR: 2.998, 95% CI: 1.566–5.739 for MFS) were valuable prognostic factors among the clinicopathologic variables examined, including sex, age, T stage, N stage, TNM stage, and differentiation.

### Association of CD161/LLT1 with the immune contexture *in situ* and in blood

**Figure 3:**
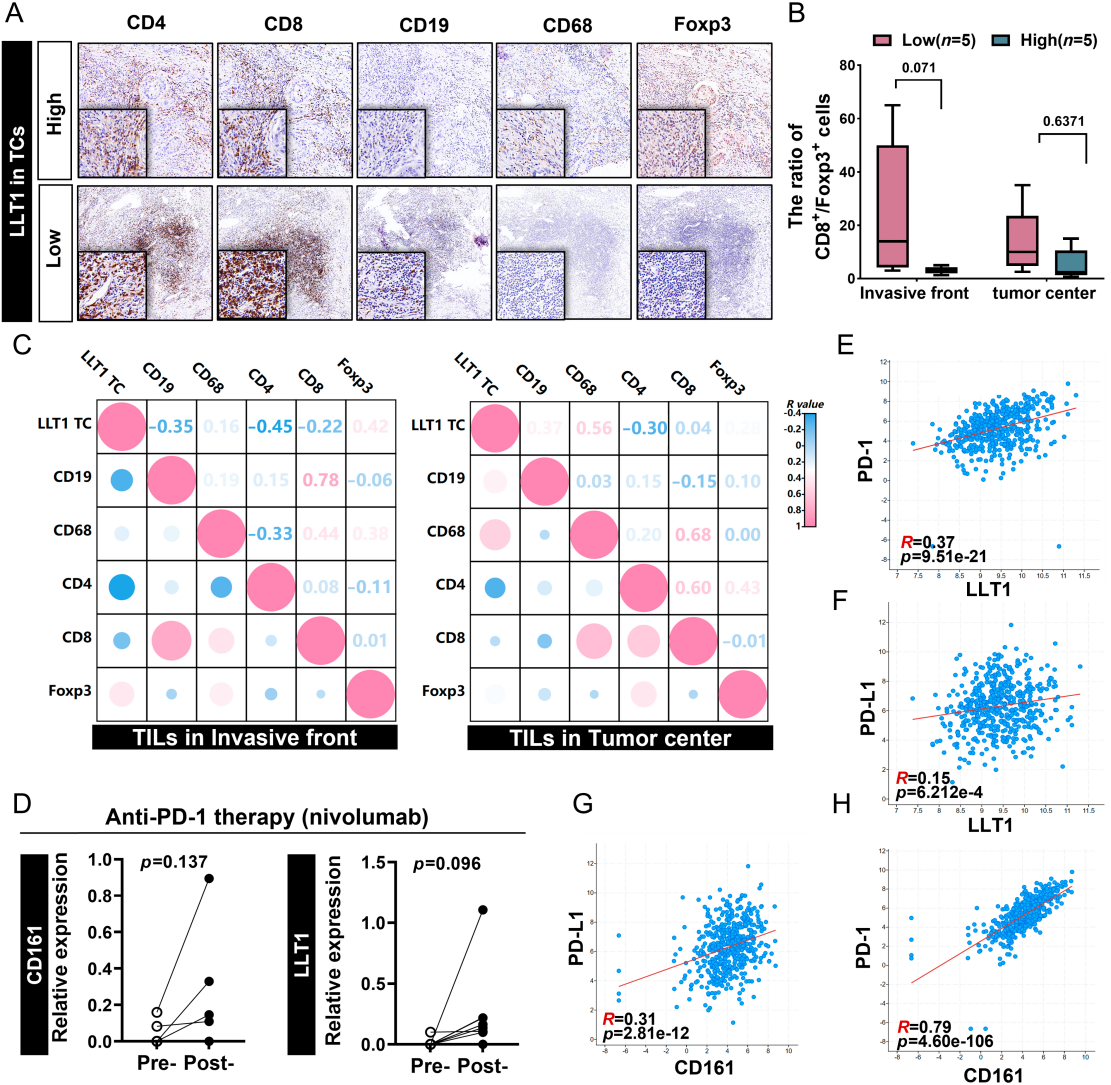
Association of CD161/LLT1 with the immune context *in situ*. (A) Typical expression images of immune markers including CD4^+^ T cells, CD8^+^ T cells, CD19^+^ B cells, CD68^+^ TAMs, and Foxp3^+^ Tregs. Each column is a different immune marker and the two rows represent the low and high LLT1^TC^ expression groups. (B) Comparison of CD8^+^/Foxp3^+^ ratios at the invasive front and tumor center between LLT1^TC^ low and high expression subgroups. Kruskal–Wallis test. (C) Heatmap showing the relationship between the expression of different cell markers, Pearson correlation analysis two tailed. (D) Analysis of the expression of LLT1 and CD161 in pretreatment or posttreatment tumors from anti‐PD‐1 therapy for resectable oral squamous cell carcinoma was analyzed (GSE179730). (E–H) Correlation between LLT1/CD161 expression and PD‐1/PD‐L1 in HNSCC with cBioPortal database. Horizontal and vertical coordinate values are the expression level of mRNA(log 2).

To delineate the immune context of LLT1^TC^ high patients, we evaluated the distribution of CD4^+^ T cells, CD8^+^ T cells, Foxp3^+^ Tregs, CD68^+^ tumor‐associated macrophages, and CD19+ B cells (Figure 3A). Fewer CD8^+^ T cells and more Foxp3^+^ Tregs were present in the high LLT1^TC^ group (tumor front: *p* = 0.071 and tumor center: *p* = 0.637; Figure 3B,C), suggesting that LLT1^TC^ high patients might have an immunosuppressive TME *in situ*.

We further analyzed the expression of CD161 and LLT1 in pretreatment and posttreatment tumors from anti‐PD‐1 therapy for resectable OSCC [31]. After PD‐1 blockade treatment, tumors showed increased CD8A expression [9], LLT1 and CD161 expression (*p* = 0.096 and *p* = 0.137; Figure 3D), indicating that they play a crucial role in the regulation of cellular immunity during PD‐1 blockade immunotherapy. Based on this finding, we investigated the correlation between CD161/LLT1 expression and PD‐1/PD‐L1 expression using the CBioPortal database. Interestingly, we found that CD161 and LLT1 were positively linked to PD‐1 and PD‐L1 (Figure 3E–H).

**Figure 4:**
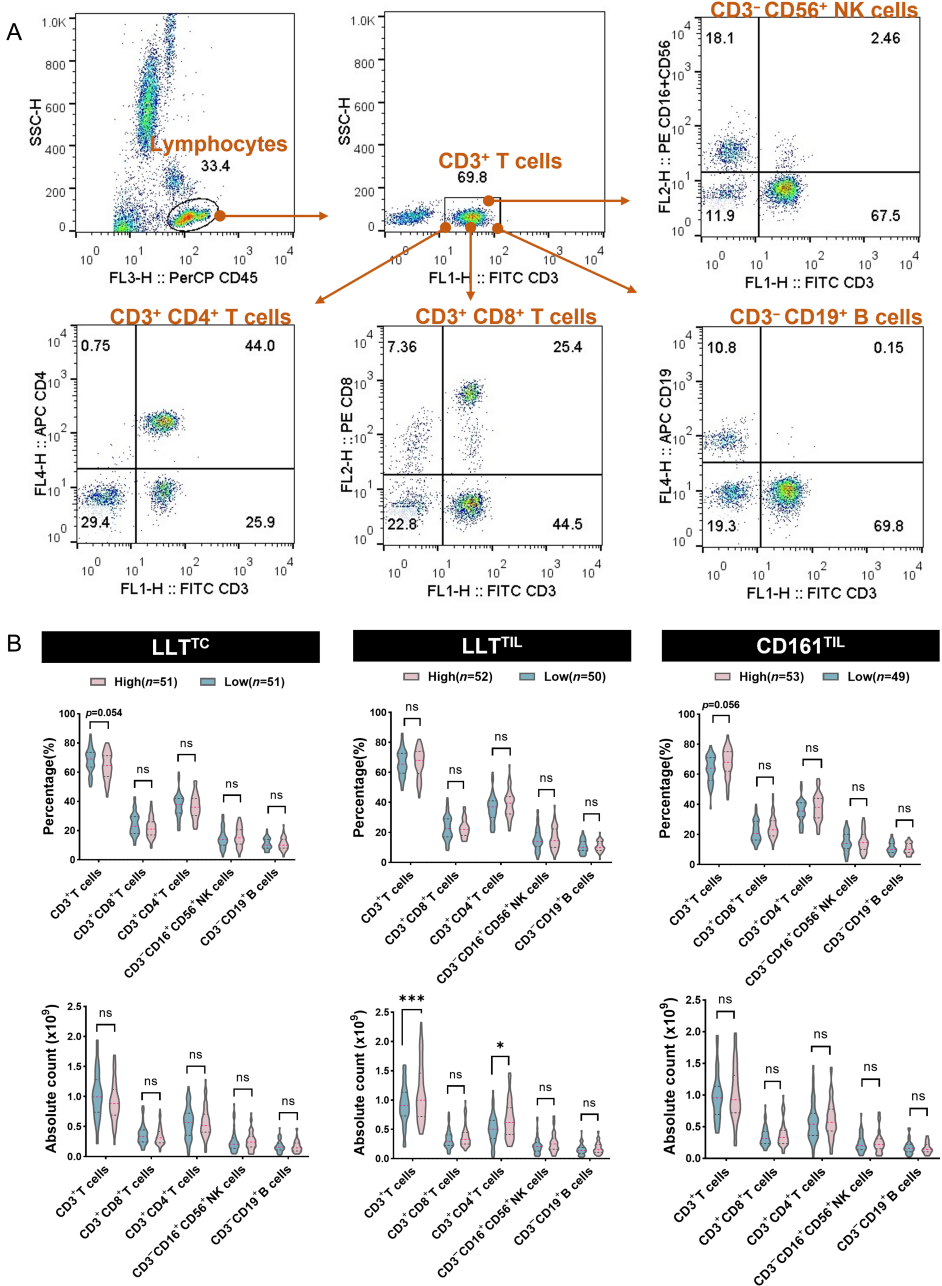
Association of CD161/LLT1 with the immune context in blood. (A) Flow cytometry analysis of lymphocytes, gated based on characteristic light‐scatter properties. Single lymphocytes were gated based on forward scatter height versus forward scatter area (FSC‐A). The numbers in the quadrants or adjacent to the lines indicate the percentage of cells. (B) Correlation of LLT1^TC^, LLT1^TIL^, and CD161^TIL^ expression in OSCC tissue with immune infiltration level (high/low) in blood before surgery. Two‐way ANOVA analysis. ns *p* > 0.05, *p ≤ 0.05, ***p ≤ 0.001.

Next, to gain a wider view of the systemic immune landscape of the 109 patient cohort, peripheral blood samples were obtained from 102 of the 109 patients and analyzed for circulating lymphocyte subsets from PBMCs (Figure 4A). Tumors containing higher LLT1^TC^ harbored a lower percent of CD3^+^ T cells (*p* = 0.054; Figure 4B), whereas those with higher CD161^TIL^ harbored an increased percent of CD3^+^ T cells (*p* = 0.056; Figure 4B). Unexpectedly, we observed that tumors with increased LLT1^+^ lymphocyte infiltration had a higher absolute count of CD3^+^ T cells as well as CD3^+^ CD4^+^ T cells (*p* = 0.0002 and *p* = 0.0118; Figure 4B), which was consistent with a previous study on oropharyngeal squamous cell carcinoma [23].

### Stratification of OSCC patients by CD161 and LLT1 level for clinical outcome prediction

**Figure 5:**
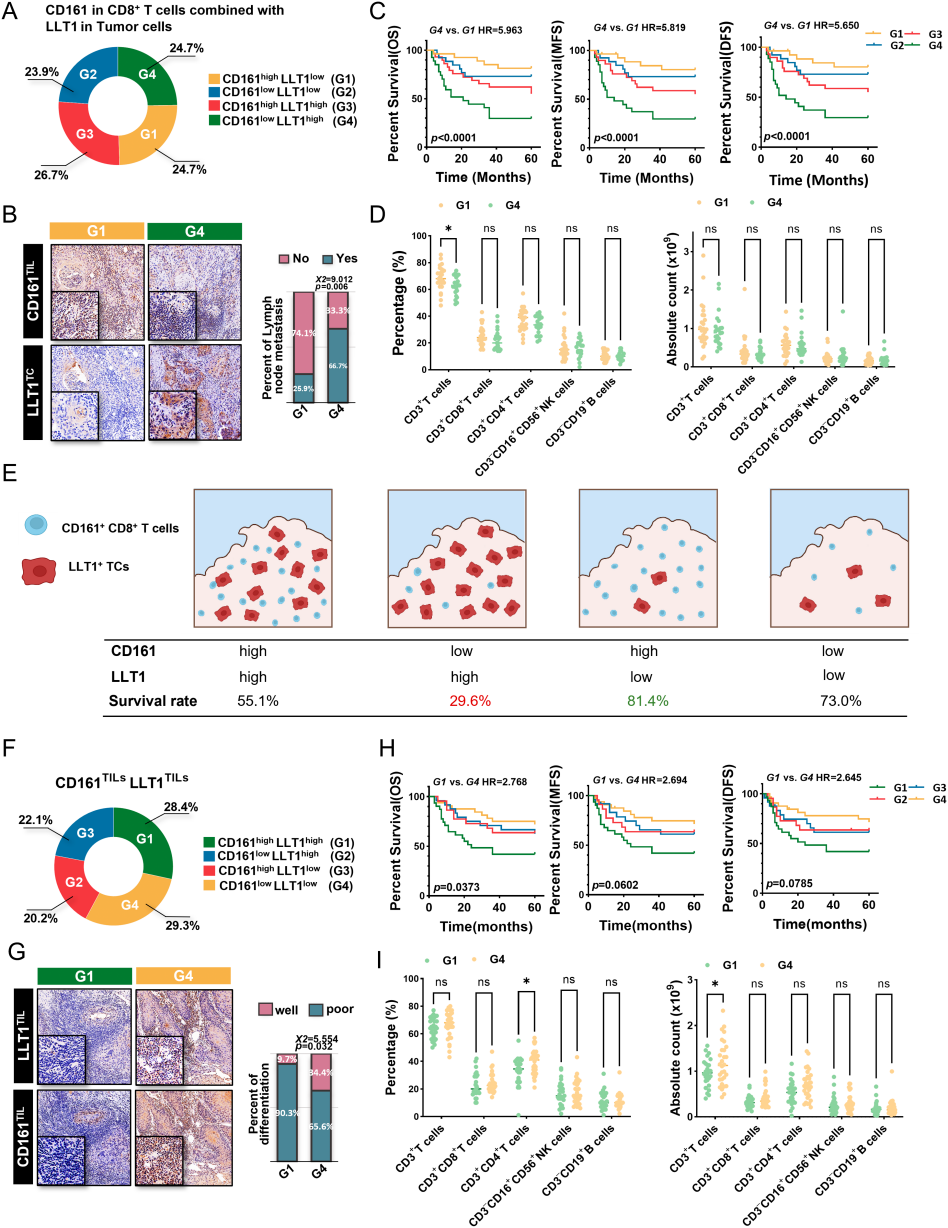
Stratification of OSCC patients by CD161 and LLT1 level for clinical outcome prediction. (A) Pie chart displaying the proportion of tumors in each subgroup based on CD161 in CD8^+^ T cells and LLT1 in tumor cells. (B) Representative images of the CD161^TIL^ high LLT1^TC^ low and CD161^TIL^ low LLT1^TC^ high subgroups. The column percentage chart shows the proportion of lymph node metastasis in these subgroups. Chi‐square test. (C) Kaplan–Meier curves of OS, MFS, and DFS according to this classification combining CD161^TIL^ and LLT1^TC^. Log‐rank test was utilized for the analysis. (D) Comparison of immune infiltration level according to the classification above before surgery. Two‐way ANOVA analysis. (E) Schematic of identification of the worst survival subgroup based on CD161^+^ CD8^+^ T cells and LLT1^+^ tumor cells. (F) Pie chart displaying the proportion of tumors in each subgroup based on CD161 and LLT1 levels in TILs. (G) Representative images of the CD161^TIL^ low LLT1^TIL^ low and CD161^TIL^ high LLT1^TIL^ high subgroups. The column percentage chart shows the proportion of well and poorly differentiated tumors in the different subgroups. Chi‐square test. (H) Kaplan–Meier curves of OS, MFS, and DFS according to this classification combining CD161^TIL^ and LLT1^TIL^. Log‐rank test was utilized for the analysis. (I) Comparison of immune infiltration level according to this classification before surgery. Two‐way ANOVA analysis. ns *p* > 0.05, *p ≤ 0.05.

Considering the known interaction between CD161 and LLT1 during T cell activation [19, 24], the CD161/LLT1 combination could predict a more accurate tumor classification for OSCC biological behavior and patient stratification compared with each biomarker alone. As the result above indicated that CD161 was mainly expressed on CD8^+^ T cells, we divided all patients into four subgroups based on CD161^TIL^ and LLT1^TC^ (CD161^low^LLT1^high^, CD161^high^LLT1^low^, CD161^high^LLT1^high^, and CD161^low^LLT1^low^; Figure 5A). Subsequently, the association between clinicopathological characteristics and survival outcomes in these four groups was analyzed further. Patients with high LLT1^TC^ expression and low CD161 expression in CD8^+^ T cells *in situ* had a higher risk of lymph node metastasis (Figure 5B) and shorter OS, MFS, and DFS (all *p* < 0.0001; Figure 5C) with reduced circulating T cells in the blood (Figure 5D). Figure 5E shows a schematic of tumor classification for OSCC biological behavior and patient stratification based on CD161^+^CD8^+^ T cells and LLT1^+^ TCs *in situ*.

Other than the expression on TCs, LLT1 was also detected in immune cells within the TME [19, 22, 23]. Thus, the patients were classified into four groups based on CD161^TIL^ and LLT1^TIL^ levels (Figure 5F). Patients whose tumors had more LLT1^+^ and CD161^+^ lymphocyte infiltration tended to have better differentiation (*p* = 0.032; Figure 5G) and a favorable survival outcome (OS: *p* = 0.0373; Figure 5H), harboring enhanced T cells in the blood (Figure 5I).

### Distinct LLT1^TC^ distribution pattern is also a risk characteristic predicting diminished T cells

**Figure 6:**
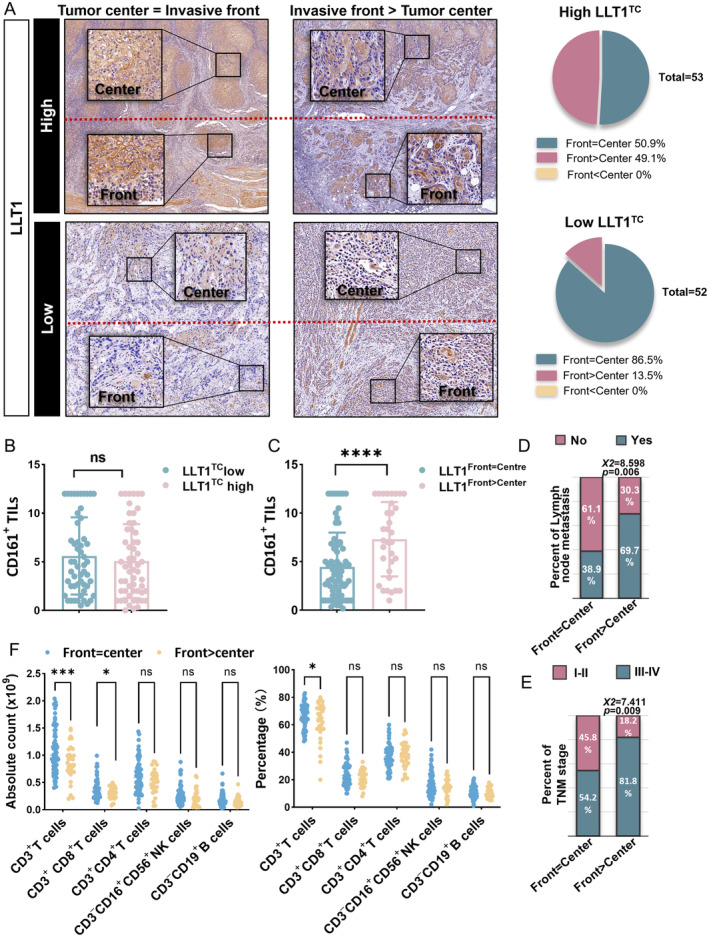
Distinct LLT1^TC^ distribution pattern is also a risk characteristic predicting diminished T cells. (A) Representative images of LLT1^TC^ expression at the invasive front and in the tumor center. The pie charts show the proportion of each subgroup based on the relationship between expression at the tumor front and in the tumor center. (B) The association between CD161^+^ immunocyte infiltration and LLT1^TC^ expression. Kruskal–Wallis test. (C) CD161^+^ immunocyte infiltration level in patients with LLT1^TC^ front >center and front = center tumors. Kruskal–Wallis test. (D) Column percentage chart showing the proportion of lymph node metastasis in different subgroups. Chi‐square test. (E) Column percentage chart showing the proportion of advanced TNM stages in the different subgroups. Chi‐square test. (F) Comparison of immune infiltration level according to the front = center and front > center classification before surgery. Two‐way ANOVA analysis. ns *p* < 0.05, *p ≤ 0.05, ***p ≤ 0.001, ****p ≤ 0.0001.

Next, when compared to that at the tumor center, we noticed that higher LLT1^TC^ levels at the tumor front were found in 13.5% of the low LLT1^TC^ group but in 49.1% of the high LLT1^TC^ group, namely ‘front > center’ (Figure 6A). Therefore, the OSCC patient cohort was divided into two subgroups according to LLT1^TC^ distribution pattern: (1) front > center and (2) front = center.

Although infiltrating CD161^+^ TILs were comparable between the high and low LLT1^TC^ subgroups (Figure 6B), patients characterized by LLT1^TC^ front > center had increased CD161^+^ lymphocyte infiltration (Figure 6C). Moreover, patients with the ‘front > center’ expression pattern exhibited a higher risk of postoperative metastasis and advanced TNM stage (*p* = 0.006 and *p* = 0.009; Figure 6D,E), and diminished CD3^+^ CD8^+^ T cells (*p* = 0.020; Figure 6F). Collectively, these results indicate that there may be a dynamic pattern of regulation between CD161 and LLT1 at the tumor invasive margin, which would lead to distinct tumor biological behavior [32].

## Discussion

Recently, many researchers have focused on the study of CD161 and its ligand, and targeting the CD161 inhibitory receptor and its ligand LLT1 could be another option for immunotherapy. In the case of gliomas, this inhibitory pathway might be of considerable significance *in vivo* and may become a therapeutic target in the future [24].

It has been previously reported that the expression of CD161 is related to a better prognosis for multiple cancers and a favorable immunotherapy response [21, 33, 34]. Conversely, one study found that, in recurrent hepatocellular carcinoma, CD8^+^ T cells overexpress CD161 and show an innate hypocytotoxic state with low clonal amplification, and that enrichment of these cells is associated with a worse prognosis [35]. Herein, we examined the expression pattern of CD161 on CD8^+^ T cells in the OSCC immune microenvironment and observed the same result that CD161^+^ TIL was a favorable prognostic indicator for OSCC. Previous studies on LLT1 have focused on lymphocytes and less on its expression in TCs. For HPV‐negative oropharyngeal squamous cell carcinoma, LLT1 was expressed in TCs, whereas the normal pharyngeal epithelium was negative [23]. In our study, LLT1^TC^ expression was higher in TCs than in TILs while high LLT1^TC^ expression was associated with a greater risk of distant metastasis and poorer survival outcomes. Instead, patients with high LLT1^TIL^ expression had a better outcome, which is consistent with previous studies [22]. Our results support that CD161/LLT1 expression may play a prominent role in OSCC progression and tumor immunity.

Recently, immunotherapy targeting the PD‐1 and its ligand PD‐L1 has provided novel strategies for the treatment of malignant tumors [7]. To better understand the altered immune microenvironment associated with anti‐PD‐1 treatment, researchers have expended considerable energy. In colorectal cancer, PD‐L1 is more often expressed on macrophages and is an M1 type of macrophage, located closer to the TCs [36]. For gastric cancer, patients with higher PD‐1^+^CD8^+^ lymphocytic infiltration have a worse prognosis and chemotherapeutic effect [37]. However, little is known about how the immune checkpoint CD161/LLT1 influences the OSCC tumor immune microenvironment. In this study, we explored the relationship between CD161/LLT1 and the immune microenvironment by examining the major immunocytes (T cells, B cells, NK cells, and macrophages). We further found that the differential expression of LLT1/CD161 led to discrepancies in the number and proportion of immunocytes *in situ* in cancer tissue as well as in peripheral blood. Another report showed that PD‐1 and CD161 were expressed on the same immunocyte subset and affected the immune environment of hepatocellular carcinoma [38]. Here, we also explored the function of CD161/LLT1 during anti‐PD‐1 treatment [31]; after therapy, tumors showed increased LLT1 and CD161 expression, and CD161/LLT1 was significantly linked to PD‐1/PD‐L1.

Considering that the CD161/LLT1 interaction would exert an influence on immune cell activation [19, 24, 25, 26], we first conducted patient stratification combining CD161 and LLT1 for more accurate assessment of OSCC biological behavior. We suggest that patients with high LLT1^TC^ and low CD161^TIL^ levels have a dismal prognosis and exhibit resistance to CD161‐based immunotherapy. Notably, we found that patients characterized by LLT1^TC^ with a front > center distribution had enhanced CD161^+^ lymphocyte infiltration. Therefore, we speculate that there is a dynamic regulatory mechanism between CD161^TIL^ and LLT1^TC^ that allows these two cells to regulate each other, as reported for PD‐1^+^ cells; these produce IFN‐γ, which stimulates the upregulation of PD‐L1 in target cells [39]. In a pioneering study, immune attack via IFN‐γ release led to inducible upregulation of PD‐L1 by mucosa, creating an ‘immune shield’ to protect against autoimmune attack in the setting of chronic inflammation or infection [39]. This is a normal cell's own protection mechanism, but is quietly exploited by cancer cells, leading to tumor immune escape. Therefore, we suggest that upregulated LLT1^TC^ at the tumor front may be due to genetic differences or an induced increase in IFN‐γ secretion by CD161^+^ lymphocytes [8, 32, 40].

In conclusion, we found that LLT1^TC^ could serve as an independent adverse prognosticator for survival in OSCC, whereas higher CD161^TIL^ and LLT1^TIL^ expression seemed to be associated with better outcomes and differentiation. In addition, the expression of CD161/LLT1 correlated strongly with the tumor immune microenvironment and PD‐1 therapy. Above all, we identify that patients with high LLT1^TC^ and low CD161^TIL^ levels have a dismal prognosis and exhibit resistance to CD161‐based immunotherapy.

## Author contributions statement

LD, XH, SX and QH contributed to data curation. YD and SX carried out formal analysis. LD, QH and XH helped with methodology. YD and XH carried out investigation. YH, YS and SX contributed to statistical analysis. LD, YN, ZW and QH supervised the study. YN helped with validation. YD, SX and XH contributed to writing the original draft.

## Supporting information


**Figure S1.** Association of CD161/LLT1 expression with clinicopathologic characteristics and prognosis
**Table S1.** Association of CD161/LLT1 expression with clinicopathologic characteristics in OSCC patients

## Data Availability

All of the data generated or analyzed in this study are included in this published article.
