# Supplementary material for: Immune checkpoint CD161/LLT1‐associated immunological landscape and diagnostic value in oral squamous cell carcinoma
Source: J Pathol Clin Res. 2024 Jan 17;10(2):e353. doi: 10.1002/cjp2.353 (PMC10792702; doi:10.1002/cjp2.353)
Supplement: Supplementary file 1 — Figure S1. Association of CD161/LLT1 expression with clinicopathologic characteristics and prognosis Table S1. Association of CD161/LLT1 expression with clinicopathologic characteristics in OSCC patients [file CJP2-10-e353-s001.pdf]

Immune checkpoint CD161/LLT1-associated immunological landscape and diagnostic value in oral squamous cell carcinoma

X Hu, Y Dong, S Xie *et al*. *J Pathol Clin Res* https://doi.org/10.1002/cjp2.353

Supplementary material

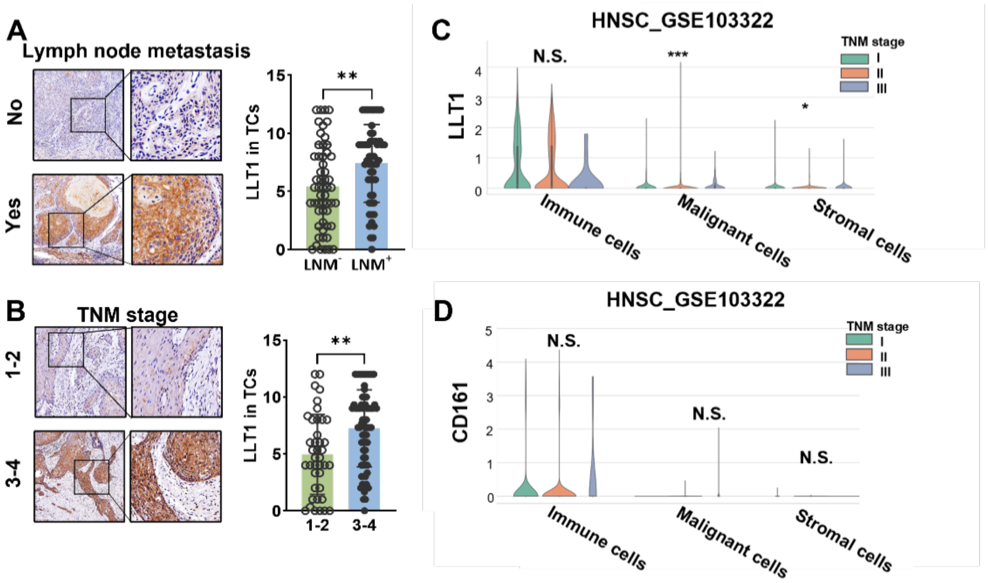

Figure S1. Association of CD161/LLT1 expression with clinicopathologic characteristics. (A, B) LLT1 expression differences between different lymph node metastasis and TNM stages in tumor cells (TCs). Mann–Whitney test. **(C)** Correlation between LLT1 expression and TNM stage in HNSCC using single-cell sequencing data (GSE103322) obtained from the TISCH2 single-cell database. **(D)** Correlation between CD161 expression and TNM stage in HNSC single cell sequencing data (GSE103322) that obtained from the TISCH2 single cell database. Kruskal–Wallis test; N.S. $p > 0.05$, * $p \leqslant 0.05$, ** $p \leqslant 0.01$, *** $p \leqslant 0.001$.

Table S1. Association of CD161/LLT1 expression with clinicopathologic characteristics in OSCC patients.

		Total N(%)	LLT1 in TC low	LLT1 in TC high	χ²	P	LLT1 in TIL low	LLT1 in TIL high	χ²	*P*
**All cases**		109(100%)								
**Gender**	Female	38(34.9%)	20(37.7%)	18(32.1%)	0.375	0.554	17(30.9%)	21(38.9%)	0.764	0.426
	Male	71(65.1%)	33(62.3%)	38(67.9%)			38(69.1%)	33(61.1%)		
**Age**	<=60	40(36.7%)	23(43.4%)	17(30.4%)	1.993	0.17	19(34.5%)	21(38.9%)	0.221	0.694
	>60	69(63.3%)	30(56.6%)	39(69.6%)			36(65.5%)	33(61.1%)		
**T stage**	Ⅰ-Ⅱ	70(64.2%)	39(73.6%)	31(55.4%)	3.937	0.071	40(72.7%)	30(55.6%)	3.497	0.074
	Ⅲ-IV	39(35.8%)	14(26.4%)	25(44.6%)			15(27.3%)	24(44.4%)		
**Lymph node metastasis**	No	55(50.5%)	35(66.0%)	20(35.7%)	10.016	**0.002***	30(54.5%)	25(46.3%)	0.742	0.446
	Yes	54(49.5%)	18(34.0%)	36(64.3%)			25(45.5%)	29(53.7%)		
**Differentiation**	Well	24(22.0%)	11(20.8%)	13(23.2%)	0.096	0.820	8(14.5%)	16(29.6%)	3.611	0.067
	Moderate to poor	85(78.0%)	42(79.2%)	43(76.8%)			47(85.5%)	38(74.4%)		
**TNM stage**	Ⅰ-Ⅱ	40(36.7%)	28(52.8%)	12(21.4%)	11.558	**0.001***	26(47.3%)	14(25.9%)	5.5345	**0.029***
	Ⅲ-IV	69(63.3%)	25(47.2%)	44(78.6%)			29(52.7%)	40(74.1%)		
			53(100%)	56(100%)			55(100%)	54(100%)		

		Total N(%)	CD161 in TC low	CD161 in TC high	X²	P	CD161 in TIL low	CD161 in TIL high	χ²	*P*
**All cases**		109(100%)								
**Gender**	Female	38(34.9%)	23(42.6%)	15(27.3%)	2.816	0.11	19(35.8%)	19(33.9%)	0.044	0.844
	Male	71(65.1%)	31(57.4%)	40(72.7%)			34(64.2%)	37(66.1%)		
**Age**	<=60	40(36.7%)	22(40.7%)	18(32.7%)	0.753	0.43	19(35.8%)	21(37.5%)	0.032	1
	>60	69(63.3%)	32(59.3%)	37(67.3%)			34(64.2%)	35(62.5%)		
**T stage**	Ⅰ-Ⅱ	70(64.2%)	36(66.7%)	34(61.8%)	0.279	0.69	33(62.3%)	37(66.1%)	0.172	0.694
	Ⅲ-IV	39(35.8%)	18(33.3%)	21(38.2%)			20(37.7%)	19(33.9%)		
**Lymph node metastasis**	No	55(50.5%)	32(66.0%)	23(35.7%)	3.316	0.086	24(45.3%)	31(55.4%)	1.105	0.34
	Yes	54(49.5%)	22(34.0%)	32(64.3%)			29(54.7%)	25(44.6%)		
**Differentiation**	Well	24(22.0%)	13(24.1%)	11(20.0%)	0.263	0.65	8(15.1%)	16(28.6%)	2.88	0.108
	Moderate to poor	85(78.0%)	41(75.9%)	44(80.0%)			45(84.9%)	40(71.4%)		
**TNM stage**	Ⅰ-Ⅱ	40(36.7%)	23(42.6%)	17(30.9%)	1.601	0.237	17(32.1%)	23(41.1%)	0.949	0.427
	Ⅲ-IV	69(63.3%)	31(57.4%)	38(69.1%)			36(67.9%)	36(58.9%)		
			54(100%)	55(100%)			53(100%)	56(100%)		
